# Composition, Structure and Diversity of Soil Bacterial Communities before, during and after Transit through the Gut of the Earthworm *Aporrectodea caliginosa*

**DOI:** 10.3390/microorganisms10051025

**Published:** 2022-05-13

**Authors:** Manuel Aira, Marcos Pérez-Losada, Keith A. Crandall, Jorge Domínguez

**Affiliations:** 1 Grupo de Ecoloxía Animal (GEA), Universidade de Vigo, 36310 Vigo, Spain; jdguez@uvigo.es; 2 Department of Biostatistics and Bioinformatics, Computational Biology Institute, Milken Institute School of Public Health, George Washington University, Washington, DC 20052, USA; mlosada323@gmail.com (M.P.-L.); kcrandall@gwu.edu (K.A.C.); 3 CIBIO-InBIO, Centro de Investigação em Biodiversidade e Recursos Genéticos, Universidade do Porto, Campus Agrário de Vairão, 4485-661 Vila do Conde, Portugal

**Keywords:** earthworm microbiome, gut transit, earthworm cast, alpha diversity, metacommunity, soil microbiome

## Abstract

Earthworms heavily modify the soil microbiome as it passes throughout their guts. However, there are no detailed studies describing changes in the composition, structure and diversity of soil microbiomes during gut transit and once they are released back to the soil as casts. To address this knowledge gap, we used 16S rRNA next-generation sequencing to characterize the microbiomes of soil, gut and casts from the earthworm *Aporrectodea caliginosa*. We also studied whether these three microbiomes are clearly distinct in composition or can be merged into metacommunities. A large proportion of bacteria was unique to each microbiome—soil (82%), gut (89%) and casts (75%), which indicates that the soil microbiome is greatly modified during gut transit. The three microbiomes also differed in alpha diversity, which peaked during gut transit and decreased in casts. Furthermore, gut transit also modified the structure of the soil microbiome, which clustered away from those of the earthworm gut and cast samples. However, this clustering pattern was not supported by metacommunity analysis, which indicated that soil and gut samples make up one metacommunity and cast samples another. These results have important implications for understanding the dynamics of soil microbial communities and nutrient cycles.

## 1. Introduction

Earthworms are key components of temperate soil ecosystems, where they constitute the largest biomass and contribute to the key processes of decomposition and nutrient cycling through their interactions with microorganisms. As detritivores and soil feeders, earthworms strongly modify the composition and structure of soil microbial communities during gut transit, which results in increased rates of microbial activity [1,2,3,4,5]. Ingestion of soil by earthworms implies that a mainly aerobic environment changes into an anaerobic one [6]. Therefore, by transiting through the earthworm gut, some microorganisms seem to perish, becoming part of the earthworm diet [7], whereas others, mainly fermenters, flourish [6,8]. Recent studies have shown that most of the fermenters found in the earthworm gut are also present in soil samples, reinforcing the tenet that the majority of bacteria found in the earthworm gut are acquired from the soil [9] and references therein. Accordingly, most of the studies characterizing the earthworm gut microbiome have revealed that its composition varies across diets and between earthworm species [1,10,11,12,13,14,15,16]. However, due to methodological limitations of the chosen techniques (PLFAs, TRFLPs, cloning and sequencing), those studies were not able to characterize in detail the composition and structure of the earthworm gut microbiome.

More recent studies using next-generation sequencing techniques have provided extensive data on earthworm microbiome composition, revealing that for earthworm casts at least, diet seems to be an important factor determining microbial composition and structure [17,18]. Moreover, it seems that earthworm casts are populated by native bacteria (i.e., bacteria not found in the earthworm diet, [19]), which contribute to the diversity of the earthworm microbiome. Since these studies were carried out in a laboratory manipulating the natural earthworm diet, it is unknown if such insights hold in natural systems, and in earthworms that feed on soil and not on decomposing organic matter. Similarly, it is still unclear what happens to the earthworm gut microbiome once egested as casts, does it change or remain unaltered? This is of critical importance since earthworm effects on soil ecosystems may occur through the interaction of casts with soil [20] or by casts’ ageing-related processes [21,22,23].

In this study, we aimed to understand how earthworms change the composition, structure and diversity of soil bacterial communities during and after gut transit. We analyzed and compared the structure of microbiomes from soil, gut and casts from the earthworm species *Aporrectodea caliginosa* (Savigny, 1826) using 16S rRNA data collected with next-generation sequencing technology. Our specific goals were to determine: (i) whether the earthworm gut microbiome is largely populated by soil bacteria or earthworm native bacteria (i.e., bacteria not present in soil), (ii) how transit through the gut modifies the composition, structure and diversity of the soil microbiome, and (iii) how these three microbiomes may be partitioned into 1–3 metacommunity types based on their taxonomic similarity. To address these questions, we chose *A. caliginosa* because it is an endogeic earthworm species (i.e., a soil feeding earthworm) and is the most prevalent earthworm in grassland and in agricultural ecosystems across temperate regions [24].

## 2. Material and Methods

### 2.1. Soil, Earthworm Gut and Cast Sampling

We sampled twenty mature specimens of *Aporrectodea caliginosa* (Lumbricidae) from a field site near to the Facultade de Bioloxía of the Universidade de Vigo by hand sorting. We also collected surrounding soil samples (0.25 g fresh weight) (0–20 cm), avoiding earthworm casts, for DNA analysis. Soil was sieved (2 mm) and placed in 20 Petri dishes. We placed earthworms individually in sterile plastic Petri dishes that were filled with that soil. Petri dishes (*N* = 20) were then randomly kept at 20 °C and 90% relative humidity in a laboratory incubator.

We sampled fresh casts by placing earthworms washed in sterile distilled water on sterile Petri dishes. Dishes were placed in the same incubator for 24 h. We handled the earthworms and dishes in a laminar flow cabinet to prevent contamination. After 24 h, we returned the earthworms to the dishes with soil and picked fresh casts with a sterile spatula, which we sterilized by flame between samplings. Sampled casts were then kept in Eppendorf tubes at −80 °C. We repeated this process up to five times to obtain 0.25 g of fresh casts per earthworm specimen (*N* = 20). We then sampled the gut of the earthworms (earthworm tissue plus gut content). To do this, earthworms were first rinsed in water, then sterilized in water, anaesthetized in diluted ethanol, placed in glass tubes with absolute ethanol and kept for two days at 5 °C until dissection. Again, all handling tasks were done under sterile conditions in laminar flow cabinet using sterilized dissection instruments (flame-sterilized between specimens).

### 2.2. Amplification, Sequencing and Analysis of 16S rRNA Genes

DNA was extracted from 0.25 g (fresh weight) of samples using the MO-BIO PowerSoil^®^ kit following the manufacturer’s protocols. DNA quality and quantity were determined using BioTek’s Take3™ Multi-Volume Plate. All laboratory procedures were performed under a laminar flow hood to prevent contamination of the samples with microorganisms from the surrounding environment.

We amplified the V4 region of the 16S rRNA gene and sequenced it following a dual-index sequencing strategy [25]. Sequencing was done on an Illumina MiSeq genome sequencer (2 × 250 bp run) at the Center for Microbial Systems, University of Michigan. Twelve gut samples did not amplify and were not included in the analysis.

We used DADA2 (v. 1.16.0) to infer the amplicon sequence variants (ASVs) present in each sample [26]. Amplicon sequence variants are more precise and reproducible than OTUs defined at a constant level (97% or more) of sequence similarity [27]. We ran trimmed and filtered forward/reverse read pairs truncating them at 220 nt and 100 nt respectively, and removed reads with ambiguous bases and more than two expected errors. ASVs were inferred from the forward and reverse of each sample, then merged. Chimeras were identified and removed when found in a sufficient fraction of the samples in which they were present. Taxonomic analysis of ASVs was carried out with RDP naive Bayesian classifier using the Silva v132 within DADA2, fixing the minBoot parameter at 80 [28,29]. Prior to ASV inference and taxonomic classification, samples had 17,228 ± 10,116 sequences. We remove ASVs unclassified at phylum level (0.5% of sequences) and remove samples that after sequence processing had less than 1000 sequences. A total of 577,510 sequences (mean: 12,287, SD: 8144) passed all quality filters and were assigned to ASVs (6217). Sequence data were uploaded to the GenBank SRA database under accession PRJNA807118.

### 2.3. Statistical Analysis

We analysed and plotted all the data in R version 3.6.1 using the phyloseq, ggplot2, ggtree and metacoder packages [30,31,32,33]. We filtered data using a prevalence criterion to keep only ASVs present in at least 2.5% of the samples. This filtering procedure removed 66% of the ASVs but only 12% of the sequences. Sampling depth was optimal for both full (6217 ASVs and 577,510 sequences, Supplementary Figure S1a) and filtered data sets (2106 ASVs and 507,459 sequences, Supplementary Figure S1b) as showed by rarefaction curves. We used the filtered data set for all statistical analysis except α-diversity estimation and metacommunity assembly.

We analyzed differential abundances of bacterial taxa applying negative binomial models using raw ASV counts as implemented in the package DESeq2 [34,35]. We tested for differential abundance of ASV and bacterial phyla among soil, gut and casts using Wald tests [34]. Since we did multiple pairwise Wald tests among the three experimental treatments (soil-gut, gut-cast and soil-cast), we adjusted “raw” *p* values using the Benjamini–Hochberg FDR method to correct for multiple pairwise comparisons. After correction, non-significant contrasts were considered to have an effect size (log2 fold change) of zero.

We defined native bacterial ASV as those ASVs present in gut and cast samples after removing ASVs present in the soil samples. We also looked for ASVs shared between soil, gut and casts in pairwise comparisons, as well as the percentage of sequences these shared ASVs comprised.

We inferred a phylogenetic tree with FastTree 2.1 [36]. We transformed raw filtered data (i.e., ASV counts) using the variance-stabilizing transformation to control homoscedasticity and unequal variances usually present in amplicon sequence data [34]. We built dendrograms (Ward method) with distance matrices (weighted and unweighted unifrac, Bray-Curtis and Jaccard) to test whether microbiomes of soil and earthworm samples (gut and cast together or alone) have the same structure. To do this, we used unifrac.weighted and unifrac.unweighted commands with 10,000 iterations [37] as implemented in mothur, and corrected obtained *p*-values with the Benjamini–Hochberg FDR correction method. We used the same procedure to test whether soil, gut and cast microbiomes differed in pairwise comparisons. These tests are best suited to hypothesis testing, as shown by Schloss [38].

We calculated taxonomic α-diversity using the number of observed ASVs as a measure of richness, and the inverse Simpson index as a measure of diversity. We used Faith’s phylogenetic diversity [39] as a measure of phylogenetic diversity. We tested the effect of the different environments (soil, gut and cast) on both taxonomic and phylogenetic α-diversity of microbiomes, using generalized linear models (GLM) [40]. We fixed error distribution and link function to reduce the deviance in the model [41]. Thus, phylogenetic diversity was analyzed using Poisson distribution and log link, while the other variables were analyzed using quasiPoisson distribution and log link. We used Tukey’s test for post-hoc comparisons, correcting *p*-values for multiple comparisons using Benjamini–Hochberg FDR as implemented in the ‘multcomp’ package [42].

Microbiomes of soil, gut and casts were clustered into metacommunities using a Dirichlet multinomial mixture model (DMM). We assessed the performance of DMM using the Laplace approximation to the negative log model [43] as implemented in mothur [44]. DMM analysis is usually applied to single time points, although it has also been successfully applied to temporal series [45], as which our data can be considered. Following Ding and Schloss [45], we ran DMM analysis on the full dataset after rarefication. We also ran it with the filtered data set after it was rarefied, to check whether prevalent filtering affected the DMM analysis output. We also studied differential abundance of ASVs between the metacommunities, using DESeq2 [34].

## 3. Results

### 3.1. Composition of Soil and Earthworm Gut and Cast Microbiomes

**Figure 1:**
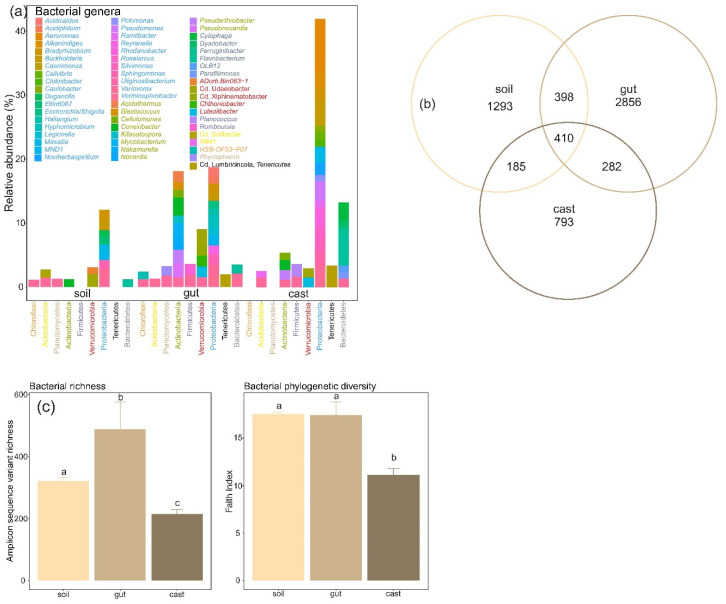
Changes in composition and diversity of soil microbiomes during and after gut transit in the earthworm *Aporrectodea caliginosa*. (**a**) Relative abundance of main bacterial phyla and genus (those with relative abundance >1%), (**b**) Venn diagram representing the number of shared ASVs between soil, gut and casts using the full data set as well as the unique ASVs of each type of sample. (**c**) Changes in taxonomic and phylogenetic α-diversity. Letters indicate significant differences between time points (Tukey HSD test).

Bacterial communities of soil, gut and casts comprised mainly bacteria from phyla Proteobacteria, Actinobacteria, Verrucomicrobia and Bacteroidetes, with minor contributions from bacterial phyla Acidobacteria, Chloroflexi, Firmicutes, Planctomycetes and Tenericutes (Figure 1a). Bacterial phyla Firmicutes and Tenericutes only appeared in gut and cast samples. For each bacterial phylum, some bacterial genera appeared in soil, gut and cast bacterial communities, including *Variovorax* (Proteobacteria), *Conexibacter* (Actinobacteria), *Flavobacterium* (Bacteroidetes), *RB41* (Acidobacteria) and *Cd*. Udaeobacter (Verrucomicrobia), whereas most were specific to each bacterial community (Figure 1a). Therefore, we found that bacterial communities of soil, gut and cast samples harboured a substantial fraction of native bacterial ASVs, i.e., ASVs that were exclusively found in one bacterial community. Bacterial communities of guts (89%) and casts (75%) were largely populated by native ASVs, although these ASVs comprised a variable fraction of sequences (65 and 34% of the sequences for gut and cast respectively, Supplementary Table S1). Bacterial communities of soil were also mainly composed of native soil ASVs (82%), although again these ASVs only comprised 42% of their sequences (Supplementary Table S1). We also found that soil, gut and cast bacterial communities shared a large proportion of their ASVs (Figure 1b). Thus, soil shared a 35% and 26% of their ASVs with gut and cast bacterial communities respectively, which comprised the 76% and 63% of soil sequences in each case. These ASVs represented 20% and 35% of ASVs from gut and cast bacterial communities, comprising 47% and 74% of their sequences. Gut and cast bacterial communities shared 17% and 41% of their ASVs, which comprised the 43% and 73% of their sequences, respectively (Figure 1b).

Earthworm samples (gut and cast together) showed a significant increase in the abundance of 59 ASVs from diverse bacterial phyla, although those with higher logFC (above 5) were from phyla Acidobacteria, (Cd. *Solibacter*), Actinobacteria (*Nakamurella*, *Kitasotospora* and *Pseudarthrobacter*), Bacteroidetes (*Cytophaga*, *Dyadobacter* and *Flavobacterium*), Firmicutes (*Paenibacillus* and Bacillales), Planctomycetes (Planctomycetales), Proteobacteria (*Aeromonas*, *Burkholderia-Caballeronia-Paraburkholderia, Chitinibacter*, Enterobacteriaceae, *Massilia*, Methylophilaceae, *Pseudomonas* and *Silvimonas*) and Tenericutes (*Cd.* Lumbricincola) (Supplementary Table S2). Earthworm samples showed significantly lower abundances than soil in 79 ASVs, which included members of phyla Acidobacteria (Acidobacteriales, and *Cd.* Solibacter), Actinobacteria (Micromonosporaceae), Armatimonadetes (Fimbriimonadaceae), Chloroflexi (Tk10), Gemmatimonadetes (Gemmatimonadaceae), Planctomycetes (Gemmataceae and WD2101 soil group), Proteobacteria (*Acidibacter*, *Anaeromyxobacter*, Burkholderiaceae, Micropepsaceae, *Rhodoplanes* and URHD0088) and Verrucomicrobia (*ADurb.Bin063-1*, *Lacunisphaera* and Pedosphaeraceae) among those with lower logFC (below −5) (Supplementary Table S2 and Figure S3).

**Figure 2:**
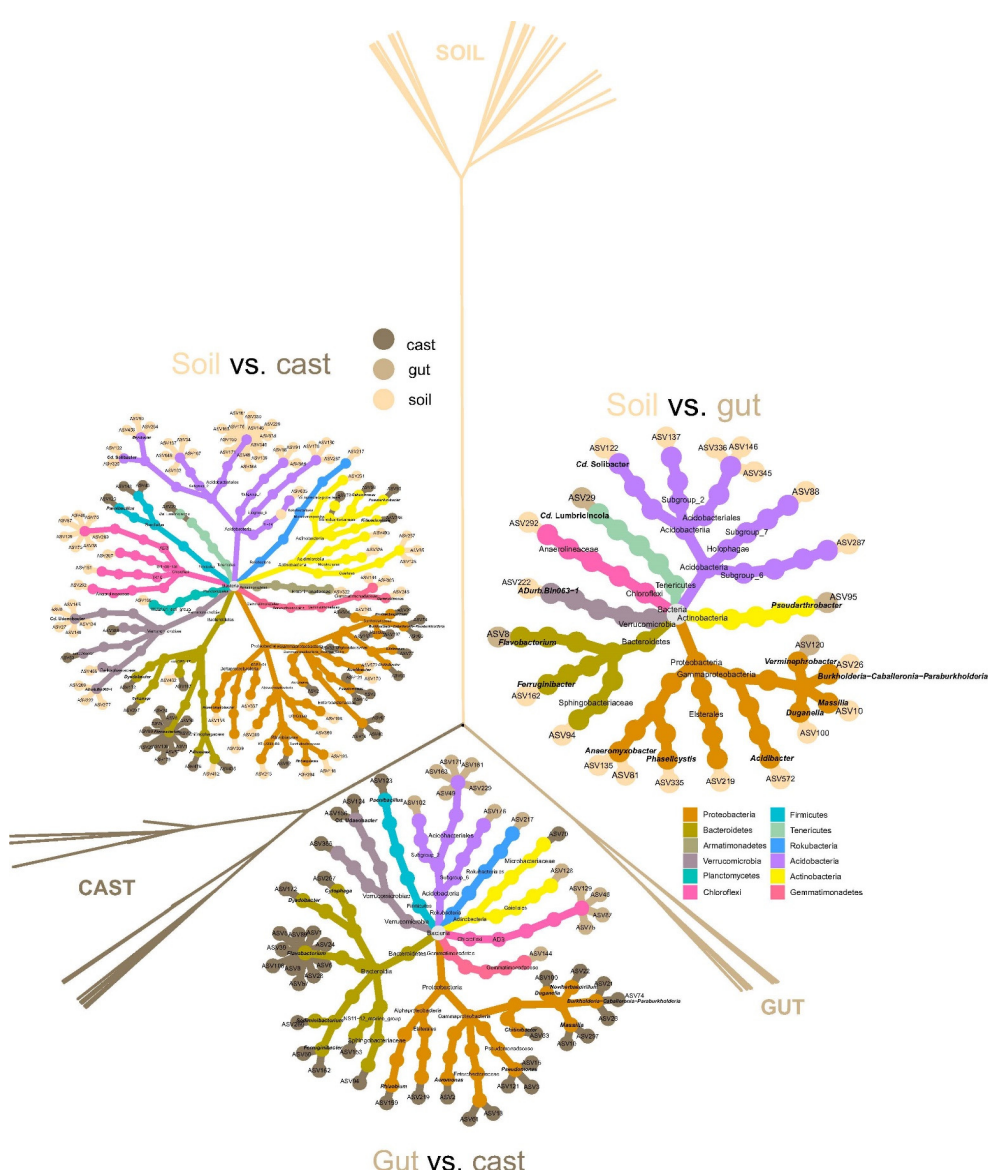
Changes in bacterial ASV abundance and structure of soil microbiome during and after gut transit in the earthworm *Aporrectodea caliginosa*. The dendrogram represents the dissimilarity of bacterial communities at ASV level (variance stabilized matrix of counts, unweighted UNIFRAC distances, Ward method). Heat trees show changes in bacterial composition across taxonomic ranges of soil samples during and after gut transit in the earthworm *Aporrectodea caliginosa*. Each tree shows bacterial ASVs with significant differential abundance after DESeq2 pairwise comparisons between soil, gut and cast samples. ASVs are colored according whether they were more abundant in soil, gut or cast. *Rhizobium* and *Burkholderia* classification are *Allorhizobium-Neorhizobium-Pararhizobium-Rhizobium* and *Burkholderia−Caballeronia−Paraburkholderia,* respectively.

Gut transit significantly increased the abundance of five bacterial phyla from soil bacterial communities, these being Tenericutes, Chlamydiae and Firmicutes, those with higher logFC (Supplementary Table S3). Bacterial ASVs showing the highest increases in abundance during gut transit (logFC > 5) were from those classified as *Cd*. Lumbricincola, *Pseudarthrobacter* and *Flavobacterium* (Figure 2 and Supplementary Table S4). Meanwhile, gut transit significantly decreased the abundance of bacterial phyla Acidobacteria, Elusimicrobia and Armatimonadetes compared to soil (Supplementary Table S3). Bacterial ASVs that showed the most pronounced decreases in abundance (logFC < −5) during gut transit were those classified as Acidobacteriales, Subgroup 6, Subgroup 2, Subgroup 8, Sphingobacteriaceae, *Ferruginibacter,* Anaerolineaceae, *Acidibacter*, *Phaselicystis*, *Duganella* and *ADurb.Bin063-1* (Figure 2 and Supplementary Table S4).

Egestion of gut microbial communities as casts resulted in significant increases of phyla Tenericutes, Bacteroidetes, Proteobacteria and Verrucomicrobia compared to gut samples (Supplementary Table S3). Meanwhile, Planctomycetes, Chloroflexi, WPS-2, Rokubacteria and Chlamydiae significantly decreased after the transition from gut to cast (Figure 2, Supplementary Table S3). Regarding bacterial ASVs, those with higher increases (logFC > 7) were classified as *Flavobacterium*, *Massilia*, *Pseudomonas*, *Chitinibacter*, *Burkholderia-Caballeronia-Paraburkholderia*, *Paenibacillus*, *Cd*. Udaeobacter, *Aeromonas*, *Cytophaga*, *Ferruginibacter* and *Dyadobacter* (Figure 2 and Supplementary Table S4). Bacterial ASVs with the most pronounced decreases (logFC > −5) were those classified as AD3, Subgroup 6, Rokubacteriales, Gemmatimonadaceae, Acidobacteriales, Gaiellales and *Cd*. Udaeobacter (Figure 2 and Supplementary Table S4).

Bacterial communities of soil after they were egested as casts showed significant increases in abundance of phyla Actinobacteria, Bacteroidetes, Firmicutes, Proteobacteria, Tenericutes and Verrucomicrobia, and significant decreases for phyla Acidobacteria, Armatimonadetes, Chloroflexi, Elusimicrobia, Gemmatimonadetes, Rokubacteria and WPS-2 (Supplementary Table S3). Bacterial ASVs showing the highest increases (logFC > 7) were those classified as *Flavobacterium*, *Cd*. Lumbricincola, *Massilia*, *Pseudomonas*, *Paenibacillus*, *Chitinibacter*, *Pseudarthrobacter* and Enterobacteriaceae (Figure 2 and Supplementary Table S4). Bacterial ASVs showing the most pronounced decreases (logFC < −7) were those classified as Subgroup 7, AD3, *Cd*. Solibacter, Subgroup 2, *Cd*. Udaeobacter, *Anaeromyxobacter*, Acidobacteriales, Gaiellales, WD2101 soil group, TK10 and Gemmatimonadaceae (Figure 2 and Supplementary Table S4).

### 3.2. Structure and Diversity of Soil and Earthworm Gut and Cast Microbiomes

The structure of soil bacterial communities significantly changed once they entered the earthworm digestive system, clustering away from the earthworm samples (gut and cast) (weighted and unweighted unifrac tests, *p* < 0.0001, Figure 1c). Moreover, earthworm digestion also significantly modified bacterial community composition, with gut and cast samples comprising two significantly different clusters (weighted and unweighted unifrac tests, *p* = 0.001, Figure 1c). The same was true for the other distance measures used (weighted unifrac, Bray-Curtis and Jaccard, Supplementary Figure S2). Transit through the gut of earthworms significantly affected the richness and diversity of soil bacterial communities. Thus, richness of gut bacterial communities was significantly higher than those of soil and cast, as it demonstrated increased diversity estimated using the inverse Simpson index (Figure 1a, Supplementary Figure S4). However, at the phylogenetic level, soil and gut samples showed the same level of diversity, whereas cast samples again showed lower phylogenetic diversity (Figure 1a).

### 3.3. Metacommunity Assembly of Soil and Earthworm Gut and Cast Microbiomes

**Figure 3:**
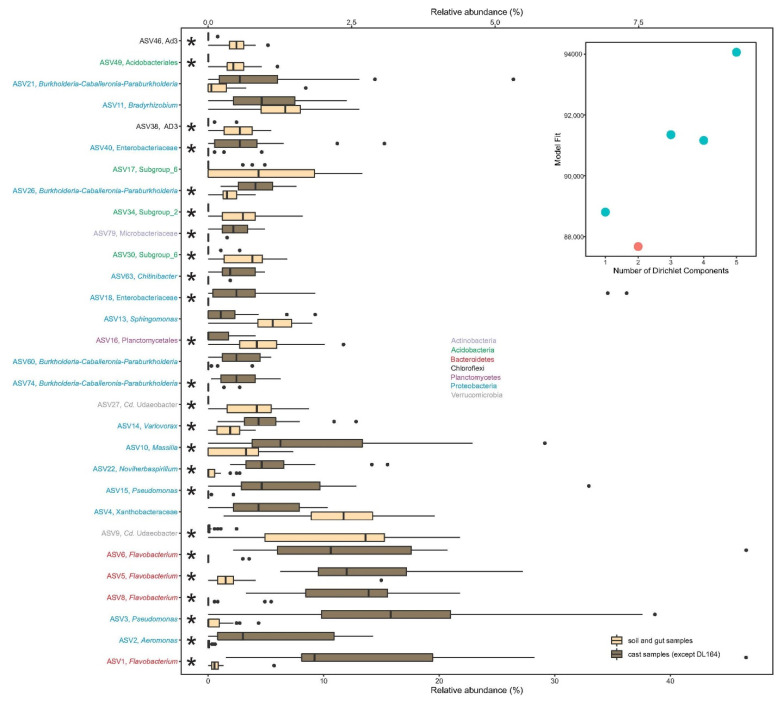
Relative abundance of the 30 most abundant ASVs in the samples assigned to each of the two metacommunity types found in transit of soil from gut to casts of the earthworm *Aporrectodea caliginosa*: metacommunity type 1 (Navajo white) corresponds to soil and gut, while metacommunity type 2 (dark brown) corresponds to cast samples. The insert represents the support for two metacommunity types when applying Dirichlet multinomial mixture models. ASVs are sorted in decreased order of importance from bottom to top. The lower *X*-axis represents ASV1 and ASV2 and the top *X*-axis the other ASVs. Asterisks indicate significant differences in ASV abundance between the two metacommunities analyzed with DESeq2. For each ASV we have included phylum (by color) and its most inclusive taxonomic classification.

Although all the dendrograms and unifrac tests indicated that soil, gut and cast samples comprise three different bacterial communities, the DMM analysis identified only two bacterial metacommunities (Figure 3, insert). These bacterial metacommunities are represented by a group of relatively abundant profiles of different ASVs (Figure 3). A close examination of the 30 most important ASVs, which accounted for 30% of the difference in fit between the two metacommunities in our data, showed that in metacommunity type 1, which included soil and gut samples, ASVs from phyla Acidobacteria (ASV30, ASV34 and ASV49), Chloroflexi (ASV38 and ASV46), Planctomycetes (ASV16), and Verrucomicrobia (ASV9 and ASV27) were significantly overrepresented, excluding ASV17 (Acidobacteria) and ASV4 and ASV13 (Proteobacteria) (Figure 3, Supplementary Table S5). On the other hand, metacommunity type 2, which included all cast samples except one, was characterized by the remaining 19 ASVs, from which ASVs from phyla Actinobacteria (ASV79), Bacteriodetes (ASV1, ASV5, ASV6 and ASV8) and Proteobacteria (ASV2, ASV3, ASV10, ASV13, ASV14, ASV15, ASV18, ASV26, ASV40, ASV63 and ASV74) had significantly higher abundances in metacommunity 2 compared with metacommunity 1 (Figure 3, Supplementary Table S5).

## 4. Discussion

As soil microbiomes pass through the earthworm gut their components experience a large change in environmental conditions, moving from aerobic (soil) to anaerobic (gut) conditions. Accordingly, facultative and strict anaerobic bacteria from soil will rise during gut transit [6]. However, there is very little, if any, knowledge of how this anaerobic microbiome returns to an aerobic one when is egested as casts. We should expect large changes in microbiome composition in the transition between gut and cast, because of reduction in water and increase in oxygen content. Furthermore, after earthworm digestion is complete, the change from metabolite concentrations that promote and feed the specific gut microbiota [6] should favor the rise of different bacterial species. Previous studies regarding the earthworm gut microbiome using next-generation sequencing techniques were focused on detritivore earthworms (i.e., earthworms that preferentially feed on decomposing organic matter), which may limit extrapolation of their results to earthworms with different feeding habits. Those studies showed that diet seems to play a prominent role in structuring the earthworm gut microbiome [17,18] and that the majority of bacteria found in earthworm casts did not come from the diet [19]. Correspondingly, although we did not test different soils, we clearly showed that the bacterial taxa populating the gut and casts of geophagous earthworms like *A. caliginosa* did not come from the diet (i.e., ingested soil). Thus, our results point to the fact that bacterial communities of earthworm guts, and hence earthworm casts, are native in earthworms in general, and that most bacteria ingested perish during gut transit.

### 4.1. Composition of Soil and Earthworm Gut and Cast Microbiomes

Composition of bacterial communities of gut and casts from the earthworm *A. caliginosa* are similar to those described for casts of this earthworm species [23] and to those of other earthworm species like *Allolobophora chlorotica*, *Aporrectodea caliginosa*, *A. tuberculata*, *Eisenia andrei*, *Eudrilus eugeniae*, *Lumbricus rubellus* and *L. terrestris*. Thus, in all cases Acidobacteria, Actinobacteria, Bacteroidetes, Planctomycetes, Proteobacteria and Verrucomicrobia comprise most earthworm gut microbiomes, with differences in abundance and composition at lower taxonomic levels that are mainly attributable to earthworm species and diet [8,9,12,17,18,19,46,47,48,49,50,51].

We found that gut and cast bacterial communities in *A. caliginosa* were populated by a majority of native bacterial ASVs, i.e., bacterial ASVs that were not found in the soil. Moreover, although a minor fraction of gut bacterial ASVs were passed to casts, these constitute a significant part of cast bacterial communities (41% and 75% of their ASVs and sequences, respectively). Our results also agree with those reported by Dominguez et al. [19] using different earthworm species and diets, but clearly contradict the tenet that most of the bacteria found in the guts of geophagous earthworms come from soil [[9] and references therein]. In fact, our data and results clearly show that soil bacteria seem to be a food source for *A. caliginosa*, at least in this soil, because we found hardly any bacterial ASV in the gut and cast samples. Soil bacterial communities also showed a high percentage of native bacterial ASVs, although they comprised barely half of the sequences. Thus, the remaining sequences belong to ASVs shared with earthworm samples, underlining the strong impact that earthworms have on soil microbial communities. Native bacteria, i.e., bacterial lineages found only in the animal and not in the environment, have been described in sponges, *Drosophila* and *Caenorhabditis elegans* [51,52,53,54], but their contribution to their gut microbiomes is lower than those described for earthworms [19].

Despite the high amount of native ASVs, we did not find many ASVs differentially abundant between soil and earthworm samples or in each pairwise soil-gut-cast comparison. However, log2 fold changes of bacterial phyla and ASVs were extremely high, with most of the values over 5 (i.e., 32 times higher), indicating the high impact that transit through the gut has on soil bacteria. As expected, gut samples showed increased abundance of Firmicutes [9,48], as well as of other bacterial phyla that exclusively thrive within earthworm guts, like *Cd*. Lumbricincola [55]. Remarkably, the presence of only one ASV of *Cd*. Lumbricincola in gut and cast samples that was absent from soil raises the question of whether this ASV is vertically or horizontally transmitted. Previous data from other known earthworm symbionts (*Verminephrobacter* and *Cd*. Nephrothrix) showed high genetic variability (i.e., a high number of ASVs) [56]. Our results support previous studies by showing that transit through the earthworm gut and earthworm casts increased the abundance of the bacterial phyla Actinobacteria, Bacteroidetes, Proteobacteria, Chloroflexy, Planctomycetes and Verrucomicrobia [19,23], although at different rates across earthworm species and diet (soil vs. sewage sludge).

### 4.2. Structure and Diversity of Soil and Earthworm Gut and Cast Microbiomes

We also found that gut transit increased the richness and diversity of the gut microbiome compared to that of the soil, but the opposite was true for the cast, which showed lower diversity values than the soil. Other studies have reported similar results, with higher bacterial diversity in earthworm samples than in their diet and values in the range of those described here [19,23,46]. However, our estimates were clearly lower than those described by Sapkova et al. [50] for *A. caliginosa*.

We found that microbiomes of soil, gut and cast showed marked and significant differences in structure. Previous studies have already shown differences in bacterial community structure between soil, gut and cast of *A. caliginosa*, although with lower resolution techniques (14). Differences between animal microbiomes and their diet or environment have been described for earthworms, sponges and nematodes [1,15,16,17,19,51,52,53,54].

### 4.3. Metacommunity Assembly of Soil and Earthworm Gut and Cast Microbiomes

Despite marked differences in composition due to native ASVs and structures, soil and gut microbiomes comprised one metacommunity, while the cast microbiome made up another. These two metacommunities were defined based on complex configurations of numerous bacterial ASVs, which were mostly over- or underrepresented in each community. This result may be due to higher levels of ASV-sharing between gut and soil than between soil and cast or between gut and cast, which was also confirmed by the amount of differentially abundant ASVs compared between soil, casts, and gut and casts together. Interestingly, these same groupings were also recovered by a dendrogram when using weighted unifrac distances, but unifrac tests did not support it.

Our results are important, because earthworm casts will enter and potentially mix with soil, modifying their bacterial communities and nutrient dynamics either by their easily assimilable nutrients [2] or by their composition [20]. In fact, we found that most of the sequences from the soil bacterial community came from bacteria shared with earthworm samples.

## Data Availability

Sequence data have been uploaded to the GenBank SRA database under accession PRJNA807118.

## Supplementary Materials

The following supporting information can be downloaded at: https://www.mdpi.com/article/10.3390/microorganisms10051025/s1, Figure S1. Rarefaction curves indicating the number of amplicon sequence variants (ASVs) identified in bacterial communities of soil, gut and casts of earthworm species *Aporrectodea caliginosa* using (a) the full dataset (6,217 ASVs and 577,510 sequences) and (b) the prevalence filtered dataset (2,106 ASVs and 507,459 sequences), Figure S2. Dendrograms representing the dissimilarity of bacterial communities at ASV level with (a) weighted unifrac, (b) Bray-Curtis and (c) Jaccard distances (variance stabilized matrix of OTU counts, Ward method). All pairwise comparisons of soil, gut and casts, as well as soil and earthworm samples (gut and cast together), were significantly different with unifrac weighted and unweighted tests, Figure S3. Variation in soil bacterial abundance through gut transit in *Aporrectodea caliginosa*. Relationship between differential ASV representation (log2 fold change) and mean of normalized counts in a) soil over earthworm samples (gut and casts samples together) with negative (Navajo white) and positive (olive drab) values indicating taxa significantly underrepresented and overrepresented in earthworm samples, respectively. (b), (c), and (d) pairwise test results for soil, gut and cast samples. In these plots, significant bacterial ASVs are colored by treatment, positive and negative logFC indicate significant increases and decreases for each treatment. In all plots non-significant taxa are colored in black, Table S1. Native bacterial ASVs from soil, gut and cast bacterial communities. Native bacterial ASVs from gut and casts are those found only in these samples and not in soil. Native soil ASVs are those found only in soil samples, Table S2. Differential abundance in bacterial amplicon sequence variants (ASVs) of soil and earthworm samples (gut and cast together) from earthworm species *Aporrectodea caliginosa* analyzed using negative binomial models, as implemented in the package DESeq2, Table S3. Differential abundance of bacterial phyla in soil, gut and casts of earthworm species *Aporrectodea caliginosa* analyzed using negative binomial models, as implemented in the package DESeq2, Table S4. Differential abundance in bacterial amplicon sequence variants (ASVs) of soil, gut and casts of earthworm species *Aporrectodea caliginosa* analyzed using negative binomial models, as implemented in the package DESeq2, Table S5. Summary of DMM analysis showing which ASVs were most responsible for separating the two communities (soil–gut and casts).
