# Supplementary material for: Composition, Structure and Diversity of Soil Bacterial Communities before, during and after Transit through the Gut of the Earthworm Aporrectodea caliginosa
Source: Microorganisms. 2022 May 13;10(5):1025. doi: 10.3390/microorganisms10051025 (PMC9144582; doi:10.3390/microorganisms10051025)
Supplement: Supplementary file 1 [file microorganisms-10-01025-s001.zip › Supplementary Figures.pdf]

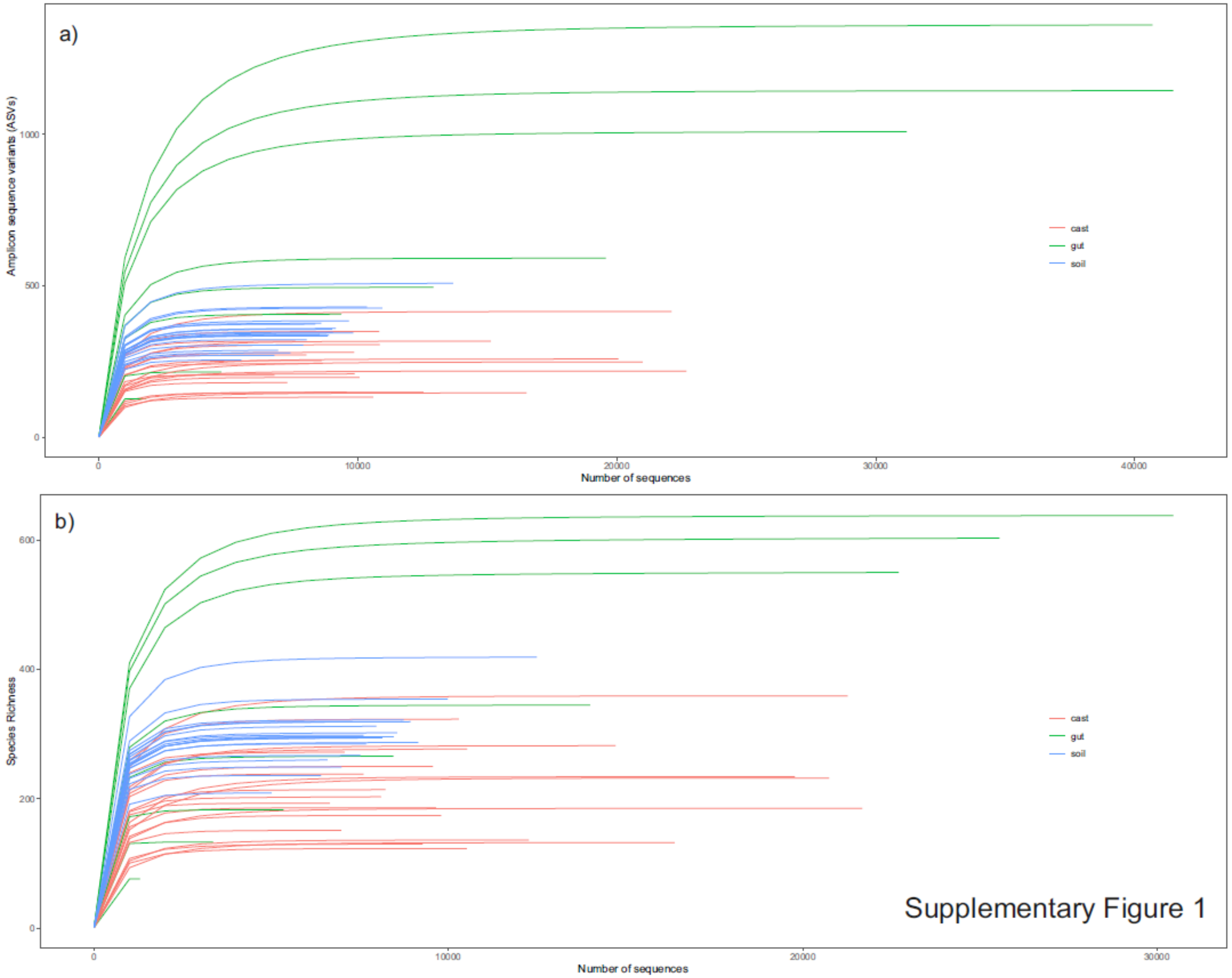

Figure S1. Rarefaction curves indicating the number of amplicon sequence variants (ASVs) identified in bacterial communities of soil and gut and casts of earthworm species *Aporrectodea caliginosa* using (a) the full dataset (6,217 ASVs and 577,510 sequences) and (b) the prevalence filtered dataset (2,106 ASVs and 507,459 sequences).

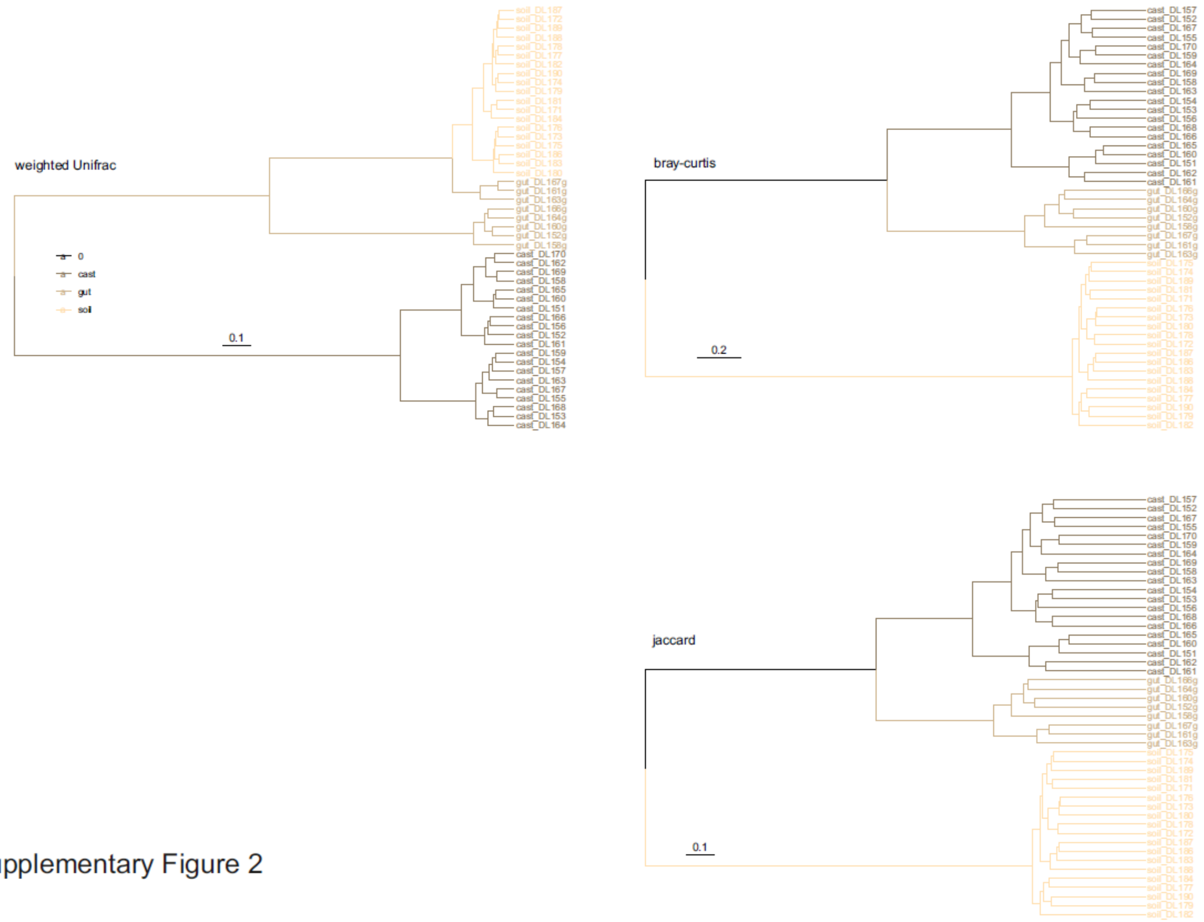

Supplementary Figure 2

Figure S2. Dendrograms representing the dissimilarity of bacterial communities at ASV level with (a) weighted UNIFRAC, (b) Bray-Curtis and (c) Jaccard distances (variance stabilized matrix of OTU counts, Ward method). All pairwise comparisons soil, gut and casts, as well as between soil and earthworm samples (gut and cast together), were significantly different with both unifrac weighted and unweighted tests.

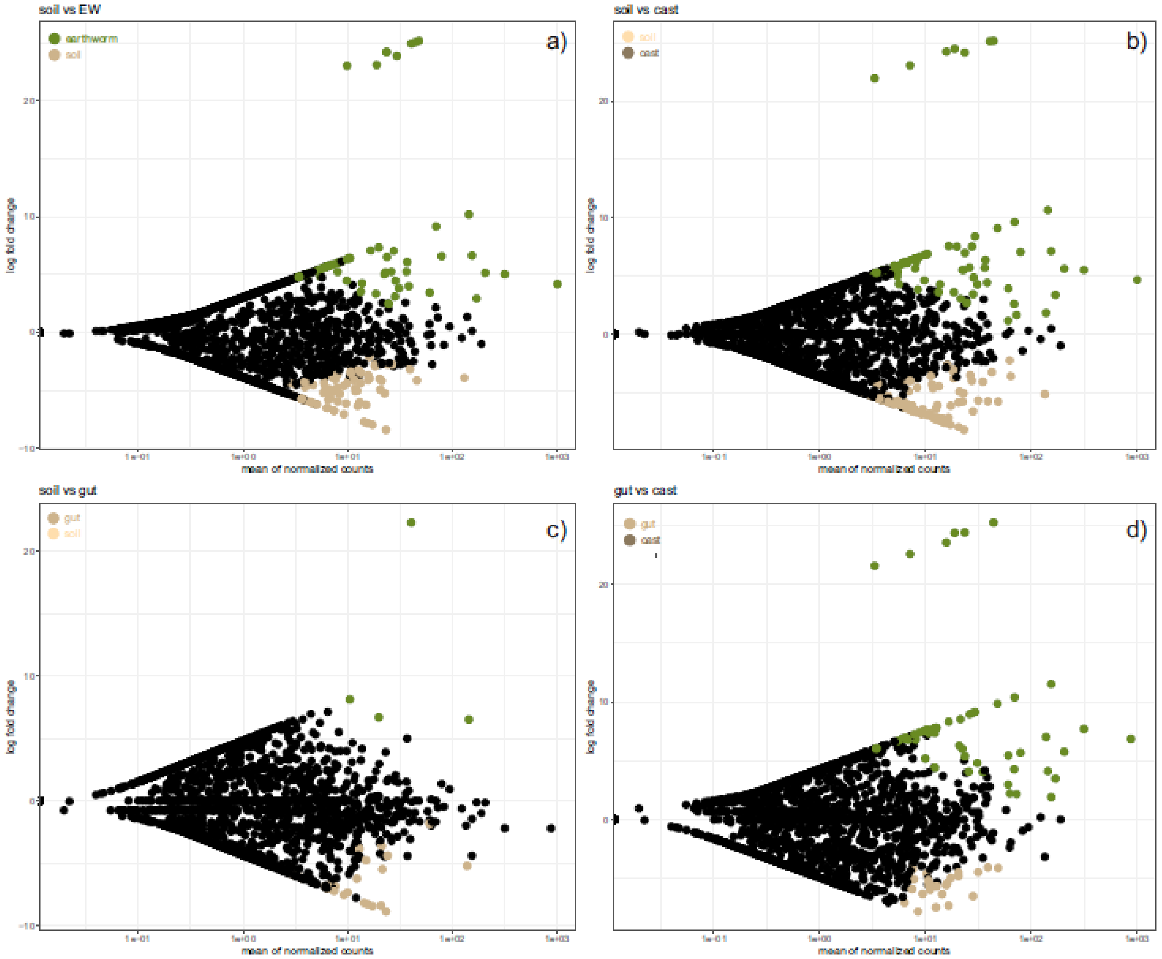

Figure S3. Variation in soil bacterial abundance through gut transit in *Aporrectodea caliginosa*. Relationship between differential ASV representation (log2 fold change) and mean of normalized counts in a) soil over earthworm samples (gut and casts samples jointed) with negative (Navajo white) and positive (olive drab) values indicating taxa significantly underrepresented and overrepresented in earthworm samples, respectively. b), c), and d) pairwise test between soil, gut and cast samples. In these plots, significant bacterial ASVs are coloured by treatment, positive and negative logFC indicate significant increases and decreases for each treatment. In all plots non-significant taxa are coloured in black.
